# The Roles of Coronary Computed Tomography Angiography in Characterizing Coronary Plaque: Screening, Treatment, and Prevention

**DOI:** 10.3390/jcdd10020043

**Published:** 2023-01-24

**Authors:** Issa Pour-Ghaz, Deya Alkhatib, Sakiru Isa, Omar Al-Taweel, Ifeoma Ugonabo, Neeraja Yedlapati, John Lynn Jefferies

**Affiliations:** 1 Division of Cardiovascular Disease, University of Tennessee Health Science Center, Memphis, TN 38163, USA; 2 Cardiology, Las Vegas School of Medicine, University of Nevada, Las Vegas, NV 89154, USA

One of the major risk factors for coronary atherosclerosis is the gradual formation and maturation of coronary atherosclerotic plaque (CAP). The primary driver of acute coronary syndrome (ACS) is the eventual instability of CAP initiating the ischemic cascade. Hyperlipidemia (HLD) is a prevalent risk factor, and despite significant recent developments in diagnosis and treatment, its management is heavily antiquated. Developments such as coronary computed tomography angiography (CCTA) have demonstrated that it is possible to detect CAP in its early stages, which, if applied to high-risk patients, can allow for the adoption of more aggressive approaches when treating HLD. Additionally, selective sequential CCTA can be applied to assess medical therapy effectiveness and assess whether an adjustment is needed prior to the development of complications. Due to the lack of such an established pathway, we propose utilizing our detection–treatment–reassessment pathway for individuals at high risk of the complications associated with coronary atherosclerosis. 

CAP is classified into calcified plaque (CP) and non-calcified plaque (NCP). The non-calcified group is the leading cause of cardiac events since it is mainly composed of cholesterol, making it vulnerable to rupture [1]. CCTA allows for the characterization of CAP; the composition of the fibroatheroma can be differentiated based on the Hounsfield units (HU) of the CP and NCP. Therefore, CCTA can identify high-risk plaques (HRP) that represent advanced complex CAP as well as those associated with higher rates of cardiac events. Based on CCTA, the four primary HRP criteria are: the napkin ring sign (NRS); low attenuation plaque (LAP); a remodeling index (RI) of >1.1; and spotty calcification of <3 mm [2,3]. NRS has an outer high-density rim (<200 HU) and an inner hypodense area (<130 HU). RI is the ratio of the vessel area at the CAP lesion segment to the vessel area at the reference segment (with positive remodeling) [2]. Coronary artery calcium scoring (CACS) is another essential tool used to risk-stratify patients; based on their Agatston score, patients are categorized as having normal (0), minimal (1–9), mild (10–99), moderate (100–399), or severe (>400) calcification [4]. However, the measured Agatston score relates to the percent of the calcified plaque and does not account for the presence of NCP, which can carry significant risk. A recent paper by Min et al. proposed a four-stage system to categorize CAP. First, CAP volume was measured as either total plaque volume (TPV) or percent atheroma volume (PAV). Then, they further characterized the CAP composition into low-density non-calcified plaque (LD-NCP), NCP, and CP. Finally, CAP could be categorized into four stages: stage 0—no plaque (TPV 0, PAV 0); stage 1—mild plaque (a mostly non-obstructive disease; TPV 0–250 mm^3^, PAV 0–5%); stage 2—moderate plaque (a combination of one vessel disease and non-obstructive disease; 250–750 mm^3^, PAV 5–15%); and stage 3—severe plaque (a multivessel disease with majority ischemic; TPV > 750 mm^3^, PAV > 15%) [5]. Other authors have used CCTA to report similar findings regarding the significance of LD-NCP and plaque characteristics with ischemia [6]. The study by the ICONIC investigators showed that through CCTA analysis, it was feasible to demonstrate volumetric differences in the composition of CAP, paving the way for the earlier detection of at-risk lesions prior to the future development of ACS [7]. When CCTA and CACS were applied to patients without known coronary artery disease (CAD) to assess their prognostic impact, it was shown that they could play a vital role in assessing NCP burden as well as long-term patient management and outcomes [8]. 

When the ability of CCTA in early recognition of CAP is considered, it naturally raises the question of whether it is possible to find any relationship between different levels of each cholesterol moiety and plaque morphology in CCTA. Manubolu et al. studied the relationship between high-density lipoprotein cholesterol (HDL-C) levels and the ratio of total cholesterol (TC) to HDL-C (TC/HDL-C) and found that HDL-C levels were inversely associated with several types of plaque findings. HDL-C levels were inversely associated with fibrous, fibrous fatty, total NC, and TPV, independent of other risk factors. They emphasize that the TC/HDL ratio is independently associated with fibrous and total non-calcified plaque even when other confounding variables are considered, underlining the significance of NCP as an etiology in ACS due to their higher risk of rupture. They also pointed out that a normal low-density lipoprotein cholesterol (LDL-C) level does not rule out other atherosclerotic abnormalities, such as a low HDL-C level, a high number of apolipoprotein B-containing particles, and small LDL-C particle size. They argued that the TC/HDL ratio could serve as an excellent marker as it provides a tremendous discriminatory ability for CAD [9]. Akin et al. studied the applicability of CCTA in the detection of CAP burden and composition and their association with serum non-HDL-C levels, triglyceride (TG), and HDL-C ratio (TG/HDL-C) in young adults (<45 years old). Their study demonstrated a significant association between non-HDL-C and TG/HDL-C ratios and CAP burden, and that the TG/HDL-C ratio was significantly associated with NCP detected on CCTA [10]. Similarly, Koide et al. evaluated the association between the TG/HDL-C ratio and HRP characteristics. They defined HRP as CAP with either positive remodeling, low-density CAP, or spotty calcifications. They found that an increased TG/HDL-C ratio, and not LDL-C levels, was associated with HRP, which can be crucial in determining the risk of future ACS. Additionally, the LDL-C level was not a strong predictor of future ACS compared to the TG/HDL-C ratio [11]. Vitamin D supplementation, a non-cholesterol variable, has also been associated with the presence of less HRP with a lower NCP burden [12]. Thus, optimizing cholesterol and non-cholesterol risk factors plays a significant role in CAD prevention. 

Despite continued efforts to optimize the management of HLD in an era of rapid advancement, which have had substantial effects on lowering lipoprotein levels, cardiovascular diseases continue to be a significant cause of morbidity and mortality. Lipoprotein(a) (Lp(a)) is a genetically determined risk factor for CAD. In a cross-sectional study of 1288 patients by Tmoyan et al., a gradual increase in Lp(a) levels was associated with a more severe atherosclerotic process involving more vascular beds. Furthermore, they found evidence that higher Lp(a) levels in combination with lower immunoglobulin M (IgM) levels, which are the “natural” antibodies produced by B1 lymphocytes, lead to a lesser ability to hinder the process of atherosclerosis, and more cardiovascular risk [13]. Recent work has further highlighted the inaccuracy in using LDL-C as a marker for starting or monitoring treatment. Shi et al. demonstrated that the level of LDL-C measured in some of the most commonly used calculations can lead to a more than 20% underestimation compared to direct measurement methods, and this was more significant in patients with high TG [14]. The atherogenic risk of TG is related to the presence of high values of remnant-C, which plays a crucial role in atherogenesis and inflammation. TG-risk lipoproteins carry cholesterol and LDL-C, while higher TG and lower HDL-C levels lead to small LDL-C particles associated with vulnerable CAP formation [11,13,15]. Thus, screening and monitoring with CCTA using an advanced lipid profile can substantially help in risk stratification and the management of a high-risk patient population. 

CCTA can help diagnose HRP early and be a guide for a more effective therapy for HLD. When appropriately utilized, CCTA can be crucial in detecting, treating, and assessing future ACS risk, especially among groups at high-risk of CAD. In Figure 1, we have outlined a flowchart that can serve as a roadmap for the optimal utilization of CCTA and laboratory testing. We believe that in the current era of CAD and HLD management, the earlier utilization of modern imaging modalities, with the help of CCTA, in conjunction with a more updated approach to measuring cholesterol levels, can help diagnose CAD in the early stages. It can also be a guide in the titration of medication to achieve optimal control. In addition, such a pathway can reduce CAD morbidity and mortality while potentially providing substantial future benefits such as reducing healthcare costs and the utilization burden. 

## Figures and Tables

**Figure 1:**
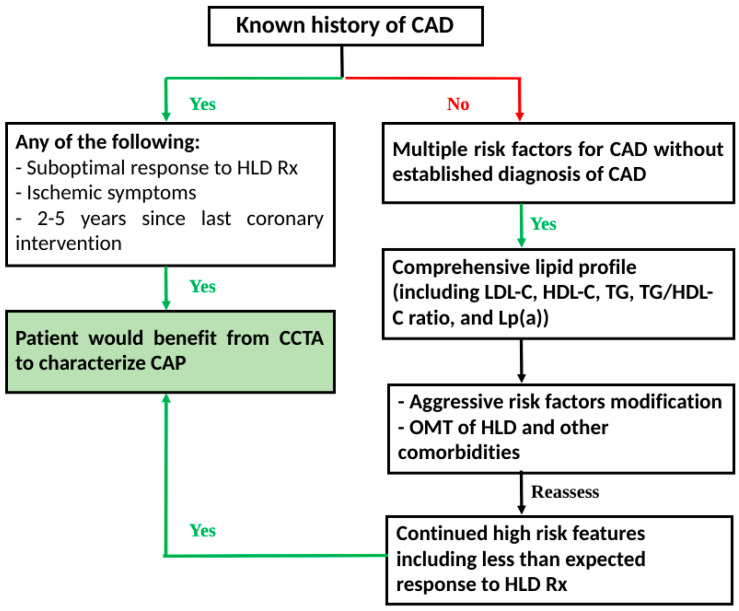
Detection–treatment–reassessment pathway: Proposed flowchart for utilization of CCTA in CAD and monitoring. CAD: coronary artery disease; CAP: coronary atherosclerotic plaque; CCTA: coronary computed tomography angiography; HDL: hyperlipidemia; HLD-C: high-density lipoprotein cholesterol: LDL-C: low-density lipoprotein cholesterol; Lp(a): lipoprotein(a); OMT: optimal medical therapy; Rx: treatment; TG: triglyceride.
